# Design and Synthesis of C-Terminal Modified Cyclic Peptides as VEGFR1 Antagonists

**DOI:** 10.3390/molecules191015391

**Published:** 2014-09-26

**Authors:** Lei Wang, Nathalie Gagey-Eilstein, Sylvain Broussy, Marie Reille-Seroussi, Florent Huguenot, Michel Vidal, Wang-Qing Liu

**Affiliations:** 1 UMR 8638 CNRS, Faculté de Pharmacie de Paris, Université Paris Descartes, Sorbonne Paris Cité, 4 avenue de l’observatoire, Paris 75006, France; E-Mails: lei.wang1@etu.parisdescartes.fr (L.W.); nathalie.eilstein@parisdescartes.fr (N.G.-E.); sylvain.broussy@parisdescartes.fr (S.B.); marie.reille@etu.parisdescartes.fr (M.R.-S.); florent.huguenot@parisdescartes.fr (F.H.); 2 UF Pharmacocinétique et pharmacochimie, hôpital Cochin, AP-HP, 27 rue du Faubourg Saint Jacques, Paris 75014, France

**Keywords:** VEGF, VEGFR, angiogenesis, cyclic peptides

## Abstract

Previously designed cyclic peptide antagonist c[YYDEGLEE]-NH_2_ disrupts the interaction between vascular endothelial growth factor (VEGF) and its receptors (VEGFRs). It represents a promising tool in the fight against cancer and age-related macular degeneration. We described in this paper the optimization of the lead peptide by C-terminal modification. A new strategy for the synthesis of cyclic peptides is developed, improving the cyclisation efficiency. At 100 µM, several new peptides with an aromatic group flexibly linked at C-terminal end showed significantly increased receptor binding affinities in competition ELISA test. The most active peptide carrying a coumarin group may be a useful tool in anti-angiogenic biological studies.

## 1. Introduction

Angiogenesis, a complex multistep process, occurs in embryogenesis, wound repair and during the female menstrual cycle. It is tightly controlled by pro- and anti-angiogenic factors and the shift of equilibrium is associated with several human diseases, as age-related macular degeneration, psoriasis, rheumatoid arthritis, or malignant tumors [1]. Its regulation depends on inhibitory and activating factors, such as vascular endothelial growth factors (VEGF). VEGFs are secreted proteins that bind to transmembrane receptors on the surface of endothelial cells (EC), inducing their dimerization, tyrosine kinase activation and the downstream serine/threonine kinase signal transduction pathways [2,3]. The VEGF receptors include VEGFR1, VEGFR2, VEGFR3, and two co-receptors, neuropilin 1 and neuropilin 2, which amplify the VEGF induced pro-angiogenic effects [4]. While VEGFR3 is involved in lymphangiogenesis, we focused on pro-angiogenic receptors VEGFR1 and VEGFR2, which are validated anticancer targets [5].

**Figure 1:**
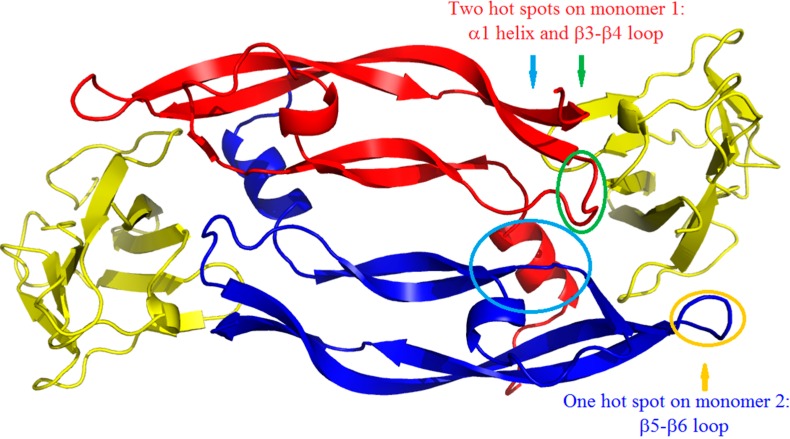
Complex of a VEGF-A dimer with two D2 domains of VEGFR1. The two VEGF-A monomers are presented in red and in blue, the two D2 are in gold. The binding sites on VEGF are circled [10].

In clinical practice, the targeting of VEGFR pathways is performed either with antibodies (for example bevacizumab, targeting the VEGF) or tyrosine kinase inhibitors (for example sunitinib or sorafenib, targeting VEGFR). Nevertheless, antibodies, which are very specific, have a high variability in their pharmacokinetic properties, while tyrosine kinase inhibitors, which have a good bioavailability and are orally active, are not specific because of the high homology among kinase domains, and constitute multi kinase inhibitors [6]. Consequently, an original approach to block the kinase activity of VEGFR, and therefore downstream kinase cascades, is to conceive VEGFR antagonists, which bind to VEGFR and compete with VEGF [7]. Such antagonists, indirectly inhibiting protein kinase activity, are in the category of inhibitors of protein-protein interactions (PPIs), a field that has drawn great interests in the last decade, leading to clinical trials of several compounds [8,9]. Our approach is based on structural data of the binding between VEGF and VEGFR (Figure 1).

VEGFR is constituted of seven extracellular domains, one transmembrane domain and a cytosolic kinase domain. Among these domains, domain 2 (D2) is the main VEGF binding site. Structures of VEGF or PlGF (placental growth factor) in complex with D2 of VEGFRs have been solved [10,11,12,13]. As can be seen on the VEGF dimer/D2-VEGFR1 complex, the binding sites concern the α1 helix and the β3-β4 loop of one VEGF monomer and the β5-β6 loop of the second VEGF monomer (Figure 1). Many efforts have been pursued in the search of antagonists of VEGF/VEGFR interactions based on the available structural data. From both the α1 helix sequence VEGF_16–25_ (KFMDVYQRSY) and the β5-β6 loop hairpin sequence VEGF_79–96_ (QIMRIKPHQGQHIGEMSF), linear or cyclic peptides and peptidomimetics have been developed [14,15,16,17,18,19,20,21]. Some of them have shown anti-angiogenic effects on *in vivo* assays and tumor growth inhibition on animal model [16,20,21,22,23], however, surprisingly, some designed peptides showed pro-angiogenic effects [21,24]. From the β3-β4 loop, we have designed in our laboratory a cyclic peptide mimicking simultaneously the β3-β4 loop and two important tyrosine residues of the α1 helix [25,26]. Some of these rationally designed peptides/peptidomimetics have been shown capable of antagonizing VEGF binding to VEGFR1. On cellular assays, they inhibit VEGF induced receptors autophosphorylation, intracellular signal pathways, such as ERK or Akt phosphorylations, and also cell proliferation and migration.

In this paper, we describe the optimization of the last β3-β4 loop (green circled site on Figure 1) derived cyclic peptide, by C-terminal modification and consequently the synthesis and biochemical evaluation on VEGFR1 binding of these new peptides.

## 2. Results and Discussion

### 2.1. Design of Peptides

In the laboratory, a series of cyclic octapeptides has been developed [25]. Such peptides, mimicking the VEGF β3-β4 loop and two aromatic residues of the α1 helix, have been shown able to compete with VEGF binding to VEGFR1. In cellular assays, these peptides inhibit VEGFR phosphorylation and downstream MAP kinases phosphorylation. They reduce HUVECs (Human Umbilical Vein Endothelial cells) proliferation and migration. NMR studies have proved that the peptide **1** interacts with the D2 domain of VEGFR1.

**Figure 2:**
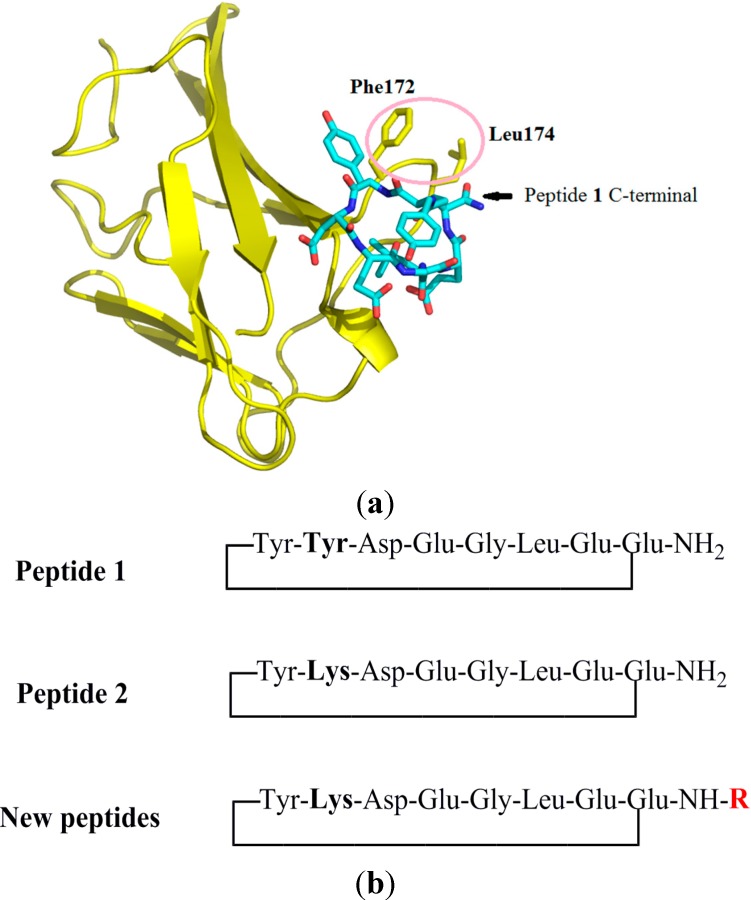
(**a**) Docking model of peptide **1** (in cyan) with the VEGFR1 D2 domain (in gold) [25]. The C-terminal amide is indicated by an arrow. (**b**) Optimization of peptide **1**. Peptide **2** with Tyr replaced by a Lys retains peptide’s receptor binding affinity but with improved solubility and creates a potential labeling site [26]. New peptides are designed with C-terminal substitutions expected to create interactions with Phe172 and Leu174 (circled in pink) belonging to the VEGFR1 D2 domain.

Manual docking followed by energy minimization of peptide **1** (c[YYDEGLEE]-NH_2_) with the VEGFR1 D2 domain is shown in Figure 2a. Two hydrophobic residues of D2 (Phe172 and Leu174) are nearby the C-terminal amide of peptide **1**. We suppose that C-terminal amide modifications might better mimic the hydrophobic Tyr25 residue of the α1 helix as in the original conception (Figure 2a). Alanine-scan and lysine-scan has shown that only the first Tyr is essential, the second one can be replaced by a Lys residue, leading to peptide **2** [26]. Although leading to a slight loss of affinity, the lysine residue in peptide **2** improves peptide solubility and provides a potential molecular labeling site as well. Moreover, peptide C- or N-terminal modifications have been proven efficient as peptide optimization strategies [27,28]. We, thus, decided to cap the C-terminal end of cyclic peptide **2** by aliphatic and aromatic groups, expecting to create new receptor binding interactions with hydrophobic residues of D2 domain, such as Phe172 and Leu174 (Figure 2a,b).

### 2.2. Synthesis of Peptides

**Scheme 1:**
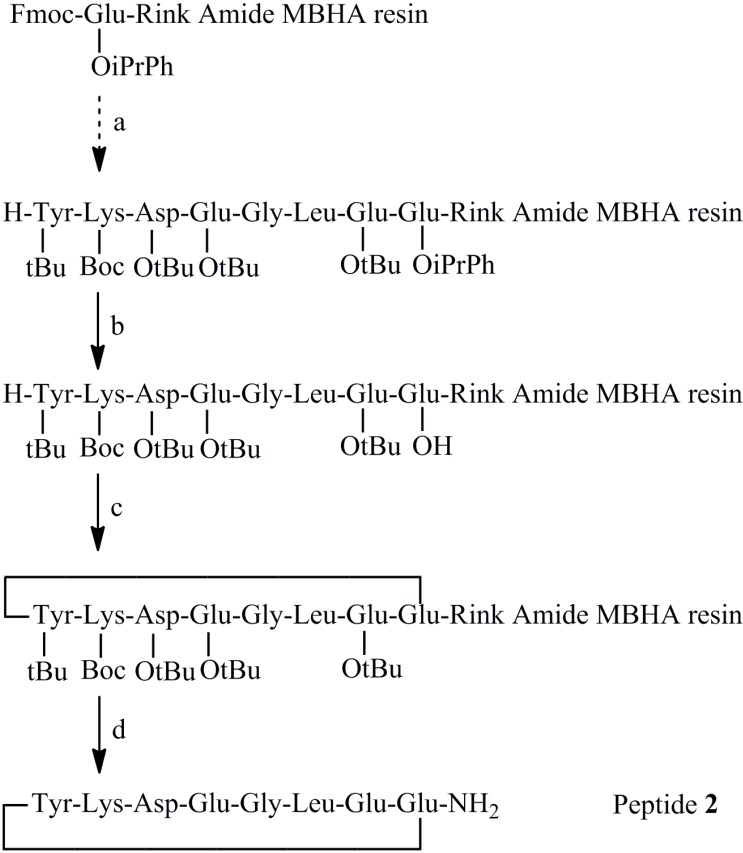
(**a**) SPPS with HBTU/DIEA coupling method. (**b**) 2% TFA with 5% TIPS in CH_2_Cl_2_. (**c**) HBTU/HOBt/DIEA in DMF. (**d**) TFA with 2.5% TIPS and 2.5% water.

Firstly, the reference peptide **2**, was prepared for comparison. In the previous synthetic route, the side chain of C-terminal Glu residue was protected in allyl ester and was removed by Pd^0^ after peptide elongation before on resin cyclisation to the N-terminal Tyr NH_2_ group [25,26]. We recently found that trace amounts of Pd might greatly influence biochemical and biological assay results [29]. Gautier *et al.* had tried using a Dmab protecting group instead of an ally group, but the Dmab could not be completely removed in the reported conditions [26,30]. We, thus, followed the same synthetic pathway to prepare the peptide **2**, but replacing the Dmab or allyl ester side chain protection with an acid labile 2-(phenyl)isopropyl (PhiPr) ester group [31] (Scheme 1). After linear peptide synthesis on Rink amide MBHA resin, the PhiPr group was removed gently by 2% TFA containing 5% triisopropylsilane (TIPS) in CH_2_Cl_2_, and cyclized by HBTU/HOBt/DIEA as described [25]. Despite the use of PhiPr protection, such on-resin cyclization encountered the problem of free amino terminus capping through guanidine formation (step c in Scheme 1) [26,32].

**Scheme 2:**
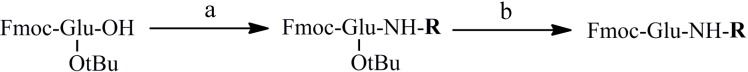
(**a**) **R**-NH_2_, HBTU/HOBt/DIEA in DMF. (**b**) 50% TFA in CH_2_Cl_2_, 1 h.

In order to synthesize the series of new peptides, we prepared modified Fmoc (9-fluorenylmethyl-oxycarbonyl) protected glutamic acids suitable for solid-phase peptide synthesis (Scheme 2).

Fmoc-Glu(OtBu)-OH was coupled with various amines by conventional 2-(1*H*-benzotriazol-1-yl)-1,1,3,3-tetramethyluronium hexafluorophosphate/1-hydroxybenzotriazole/diisopropylethylamine (HBTU/HOBt/DIEA) coupling reagents in N,N-dimethylformamide (DMF), then the side chain *t*Bu ester was deprotected by 50% trifluoroacetic acid (TFA) in dichloromethane to give the desired Fmoc-Glu-NHR in good overall yields (50%–90%). The commercially non-available amine 4-(aminomethyl)-7-methoxy-2*H*-chromen-2-one was prepared following the described Deléphine method for the 6,7-dimethoxycoumarin analog [33]. It has been reported that the Fmoc group is sensitive to organic bases and can be removed, not only by secondary amines, such as piperidine, but also by primary amines, such as cyclohexylamine and ethanolamine, especially in the polar solvent DMF [34]. Thus, the Fmoc-Glu-(OtBu)-OH was preactivated 30 min by HBTU/HOBt/DIEA before the addition of 1.8 equivalents of amine.

**Scheme 3:**
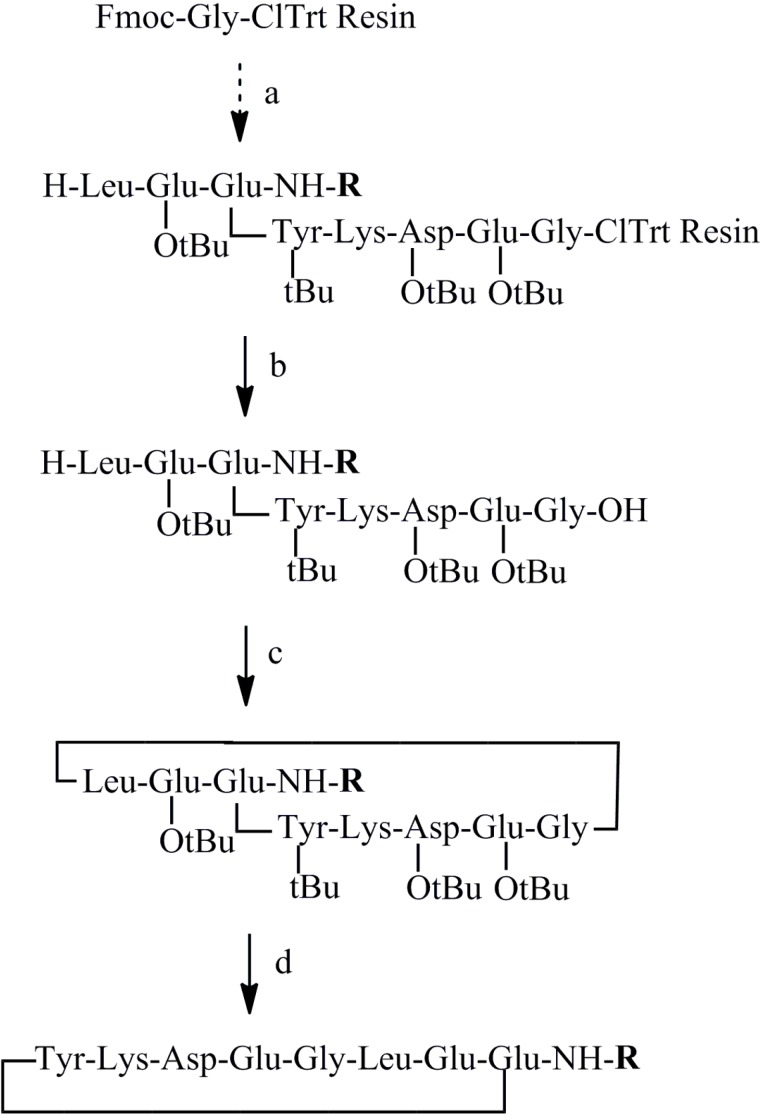
(**a**) SPPS with HBTU/DIEA coupling method. (**b**) 2% TFA with 5% TIPS in CH_2_Cl_2_. (**c**) DIC/HOAt in DMF 1–3 days. (**d**) 50% TFA in CH_2_Cl_2_ 2 h.

Because the series of new peptides designed has the C-terminal amide capped, the same synthetic pathway cannot be applied. Although it is possible to envision on-resin cyclization by loading the resin on the side chains of Asp or Glu in the peptide sequence, we decided to realize the cyclization in the solution phase between two small amino acid residues Gly/Leu [35], to minimize the guanidine formation cited above (Scheme 3). 

Peptides were synthesized with microwave irradiation during coupling and Fmoc-deprotection. Starting from acid labile 2-chlorotrityl resin, the first Fmoc-Gly residue was loaded in the presence of DIEA, the subsequent Fmoc-protected amino acids were introduced stepwise. Due to the instability of the 2-chlorotrityl resin, the synthesis was programmed with weak microwave irradiation (50 °C). HBTU/DIEA was used as coupling method to keep the acid sensitive resin in basic conditions. This classic method gave better results than a more recently developed diisopropylcarbodiimide (DIC)/OxymaPure coupling method [36], since OxymaPure is a weak acid (pKa 4.6) and may slightly cleave the peptide from the chlorotrityl resin at each coupling step [37]. After the final Fmoc deprotection, the side chain-protected peptides were freed from resin by 2% TFA, then cyclized in DMF with DIC/1-hydroxy-7-azabenzotriazole (HOAt) (3 eq each) as coupling agents. The cyclisation was checked by HPLC analysis. After completion, generally overnight or two to three days, DMF was removed by evaporation and the peptide was precipitated in water and washed thoroughly with aqueous NaHCO_3_ solution to removed diisopropylurea and HOAt. Then, the peptide was fully deprotected by 50% TFA in the presence of 2% of TIPS and purified by HPLC. Generally, this synthetic pathway gives satisfactory total yield.

### 2.3. Evaluation of the Inhibitory Effect of Peptides on the VEGF-VEGFR1 Interaction

Peptides VEGFR1 binding ability was determined by a competition ELISA test [38]. Briefly, recombinant human VEGFR1 extracellular domains (ECD) were adsorbed on the surface of a 96-well microplate. After washing and BSA blocking, the plate was incubated with different compounds at 100 µM in competition with biotinylated VEGF (btVEGF). After additional washing steps, the remaining btVEGF was detected by chemiluminescence, via an HRP-conjugated streptavidin. The results in Table 1 are presented in percentages of VEGF displaced compared to the value without peptide competitor.

**Table 1:** Cyclic peptides with C-terminal substitutions. Displacement represents the percentage of btVEGF displaced by the peptides at 100 µM. The values are the average of at least 3 tests each in triplicate. 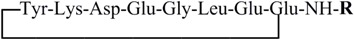

Peptide	R	Displacement 100 µM (%)	Peptide	R	Displacement 100 µM (%)
**2**	H [26]	12	**11**	-CH_2_-CH_2_-CH_2_-Ph	12
**3**	pentyl	19	**12**	-CH_2_-CH(Ph)_2_	20
**4**	isobutyl	11	**13**	(1-naphthalene)methyl	24
**5**	allyl	35	**14**	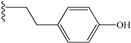	40
**6**	(2-hydroxy)ethyl	23	**15**	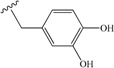	58
**7**	cyclohexyl	9	**16**	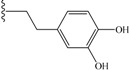	53
**8**	(cyclohexyl)methyl	7	**17**	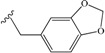	42
**9**	-CH_2_-Ph (benzyl)	39	**18**	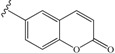	14
**10**	-CH_2_-CH_2_-Ph	43	**19**	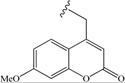	68

Addition of aliphatic groups at the C-terminal position had little effect on the peptide’s receptor binding affinity (peptides **3**–**8**). Sterically hindered groups like cyclohexyl decreased the percentage of VEGF displaced. However, allyl or hydroxyl groups, more electron-rich, increased slightly the value (peptides **5** and **6**).

Among the aromatic substituents, benzyl groups linked through one or two methylene were beneficial (peptides **9** and **10**), but a three methylene linker was detrimental (peptide **11**). Two phenyl groups (peptide **12**) showed also a weak effect. These results suggest that the targeted pocket believed to interact with the C-terminal group is near as expected, but not big or deep. Following the result of peptide **6**, we then were interested by hydroxyl substitution on the phenyl group. Hydroxy or oxygen ester/ether substitutions were beneficial for receptor binding. Although peptide **14** showed similar affinity as peptide **10**, peptides **15** and **16** were, effectively, much more active. We suppose that because a hydroxyl group is both a hydrogen bond acceptor and donor group, it may create new interactions with some receptor residue especially when it is at the *meta* position. This is supported by the fact that cyclization of two hydroxyl groups in peptide **15** by a methylene decreased the affinity (peptide **17**). As in the case of aliphatic substitutions, direct aromatic substitution decreased greatly the peptide’s affinity (peptide **18**). However, when the coumarinyl group was linked by a rotable methylene (peptide **19**), we recovered the peptide’s affinity, suggesting both a hydrophobic pocket on the VEGFR1 D2 domain and the importance of hydrogen bond.

We have to stress here that the commercial btVEGF does not have the same VEGF biotinylation level for different batches. The quantity of btVEGF used for each assay must be verified in order to reach the same signal level (in relative light units). If the VEGF biotinylation level is low, we have to introduce more btVEGF (containing unbiotinylated VEGF), which may give a lower peptide inhibition value. Thus, peptide **2** did not give the same displacement value as previously reported (45% in reference 26). Peptide **1** in reference 25 and 26 did not have the same inhibition (57% and 73% respectively). All the peptides have to be compared using the same btVEGF batch, which is the case in Table 1. The determination of IC_50_ of these peptides by ELISA revealed uncertain values. The problem was also observed by the group of Barker who works on an anti-angiogenic peptide derived from tissue inhibitor of metalloproteinases-3 (TIMP-3) capable of binding to VEGFR2 ECD [39]. In their study, incomplete btVEGF displacement has also been obtained in their ELISA assay. The authors suggested that increasing inhibitor in high concentrations led to non-specific binding of btVEGF to the plate. To confirm that our peptide’s affinity is not a non-specific binding, peptides **15**, **16**, and **19** were assayed at three different concentrations (Table 2).

**Table 2:** Dose dependent inhibition of selected peptides on VEGFR1 binding measured by ELISA. The values are the average of at least 3 tests each in triplicate. NA means no activity.

Peptide	Displacement (%)		
	100 µM	50 µM	30 µM
**15**	58	37	14
**16**	53	31	NA
**19**	68	41	11

Indeed, all three peptides dose dependently inhibit VEGF/VEGFR1 binding, highlighting a targeted VEGFR1 binding. Over 100 µM, peptides gave sometimes-lower btVEGF displacement due to peptide precipitation and aggregation in addition to the non-specific btVEGF effect.

Initial reference peptide **1** had been studied by NMR in complex with the D2 domain of VEGFR1 and had been proved to share with VEGF [10] a common binding site on D2 (His223, Arg224) [25]. Moreover, immobilized peptide **2**, through the lysine side chain, can pull down VEGFR1 D2, VEGFR1 ECD, and VEGFR2 ECD, and do not bind to VEGF co-receptor neuropilin 1 [26]. In fact, neuropiline 1 binds to the extreme C-terminal domains (exons 7 and 8 encoded) of VEGF_165_, the dominant isoform responsible for pathological angiogenesis, while VEGFR1 and VEGFR2 bind to the domains encoded by exons 2–5 [40]. Based on the results obtained in References [25] and [26], we believed that our new cyclic peptides do mimic VEGF to interact with the domain D2 of VEGFR. Peptides **15**, **16**, and **19** with improved receptor binding affinities in this study are now in further structural studies and cellular evaluations. We cannot yet define their binding pocket on D2, but we suppose that this C-terminal group may establish new interactions with Leu174 and Phe172 of the D2 domain by hydrophobic and hydrogen bond interactions. A detailed NMR study is underway to confirm this hypothesis and validate the conception. Peptide **19**, carrying a hydrophobic fluorescent coumarinyl group at the C-terminal end, although slightly less water soluble than **15** and **16**, will be very helpful in co-crystallization and biological imaging studies.

## 3. Experimental Section

All conventionally protected amino acids, peptide synthesis reagents and organic solvents were from Carlo Erba Reagents (Val de Reuil, France) and other chemical products from Sigma Aldrich (St. Louis, MO, USA) or Alfa Aesar (Ward Hill, MA, USA). Fmoc-Glu(O-2-PhiPr)-OH was from Novabiochem^®^ (Merck Millipore, Darmstadt, Germany) ^1^H-NMR spectra were recorded on a Bruker spectrometer (300 MHz) and were internally referenced to residual protonated solvent signals. Mass spectrometry spectra were recorded on a Waters ZQ 2000 spectrometer. Peptides were synthesized in solid phase using Fmoc chemistry on microwave assisted CEM-Liberty 1 synthesizer. DMF was used as solvent. Crude peptides were purified on a Waters 600 semi-preparative HPLC system using a GRACE Vydac Protein and Peptide 218TP column (10 × 250 mm) with a linear A-B gradient at a flow rate of 2 mL/min. Mobile phase A was 0.1% TFA aqueous, and B was 0.09% TFA in 70% acetonitrile aqueous solutions. Peptides were analyzed on a Shimadzu Prominence LC-20AD HPLC using a GRACE Vydac Protein and Peptide 218TP column (4.6 × 250 mm) with a linear A-B gradient at a flow rate of 1 mL/min where solvents A and B were as described above. Absorbance signals of peptides were detected at 214 nm. The purity of the peptides was verified by analytical HPLC as described above, and the purified peptides were further characterized by mass spectrometry.

### 3.1. Preparation of 4-Aminomethyl-7-methoxy-chromen-2-one Hydrochloride

4-Bromomethyl-7-methoxy-chromen-2-one (0.84 g, 2.7 mmol) was dissolved in 30 mL chloroform, hexamethylenetetramine (0.57 g, 4.0 mmol) was added and the mixture was stirred at room temperature during 24 h. The resulting precipitate was filtered, washed with CHCl_3_ dried to yield 1-[(7-methoxy-2-oxo-2*H-*chromen-4-yl)methyl]-3,5,7-triaza-1-azoniatricyclo[3.3.1.13,7]decane bromide as a yellow solid. This intermediate was then dissolved in 20 mL ethanol containing 3 mL of concentrate HCl (37%) and refluxed until the yellow mixture gradually turned white, indicating the completion of the hydrolysis. The reaction mixture was cooled and the precipitate was collected by filtration. The white solid was washed with ethanol and dried to give 4-aminomethyl-7-methoxy-chromen-2-one hydrochloride (0.29 g, yield 53.5%). Its spectroscopic and analytical properties were identical to those reported [41]. ^1^H-NMR (DMSO-d_6_): δ 3.87 (s, 3H, OCH_3_), δ 4.35 (s, 2H, CH_2_-N), δ 6.44 (s, 1H, H-3), δ 6.95 (dd, 1H, H-6), δ 7.00 (d, 1H, H-8), δ 7.51(d, 1H, H-5), δ 9.06 (s, 3H, NH_3_^+^).

### 3.2. General Method for the Preparation of Substituted Glutamic Amides (Compounds **1**–**17**)

Fmoc-Glu(OtBu)-OH (1.28 g, 3 mmol) was dissolved in 15 mL DMF, HBTU (1.36 g, 3.6 mmol), and HOBt (0.48 g, 3.6 mmol) were added. After complete dissolution, DIEA (1.05 mL, 6 mmol) was added and the mixture was stirred at room temperature 30 min before addition of R-NH_2_ (5.4 mmol). The completion of the reaction was checked by TLC. Then DMF was evaporated *in vacuo* and the residue was triturated in water to give a precipitate, which was thoroughly washed successively with 10% Na_2_CO_3_ solution, 10% citric acid solution, and water. If the product was not solidified, the residue was taken in ethyl acetate and washed successively with 10% Na_2_CO_3_ solution, 10% citric acid solution, and water, dried and evaporated to give the crude Fmoc-Glu(OtBu)-NHR. Generally, without further purification, the crude Fmoc-Glu(OtBu)-NHR was treated with 50% TFA in CH_2_Cl_2_ containing 2.5% TIPS at room temperature during 1 h. After removal of TFA and solvent, the residue was solidified in ether/cyclohexane and collected by filtration. Purification by chromatography on silica gel with CH_2_Cl_2_ containing 1%–5% of methanol and 0.1% of acetic acid gave Fmoc-Glu-NHR for further peptide synthesis. Trace amounts of acetic acid must be removed by lyophilization of the final product’s aqueous suspension before being used in peptide synthesis, to avoid peptide acetyl capping. The total yields were 50%–90% in two steps.

^1^H-NMR chemical shifts for Fmoc protected glutamic amide are in ppm. The black values are for various R groups, and gray italic for the same Fmoc and glutamic core groups.

*Fmoc-Glu-NH-(CH_2_)_4_CH_3_* (**1**): yield 94%. ^1^H-NMR (DMSO-*d*_6_): 0.8 (t, 3H, CH_3_), 1.2 (m, 4H, 2 × CH_2_), 1.4 (m, 2H, CH_2_), 3.90 (m, 2H, CH_2_-N), 7.85 (d, 1H, NH). 1.8 (m, 2H, CH_2_β), 2.2 (m, 2H, CH_2_γ), 4.0 (m, 1H, CHα), 4.3 (m, 3H, 9-H, CH_2_Fmoc), 7.32 (t, 2H, H_Ar_Fmoc), 7.4 (t, 2H, H_Ar_Fmoc), 7.5 (d, 1H, NH), 7.75 (d, 2H, H_Ar_Fmoc), 7.9 (d, 2H, H_Ar_Fmoc), 12.1 (s, 1H, CO_2_H).

*Fmoc-Glu-NH-CH_2_CH(CH_3_)_2_* (**2**): yield 82%. ^1^H-NMR (DMSO-*d*_6_): 0.8 (d, 6H, 2 × CH_3_), 1.7 (m, 1H, CH), 2.9 (m, 2H, CH_2_-N), 7.85 (d, 1H, NH). 1.8 (m, 2H, CH_2_β), 2.2 (m, 2H, CH_2_γ), 4.0 (m, 1H, CHα), 4.3 (m, 3H, 9-H, CH_2_Fmoc), 7.32 (t, 2H, H_Ar_Fmoc), 7.4 (t, 2H, H_Ar_Fmoc), 7.5 (d, 1H, NH), 7.75 (d, 2H, H_Ar_Fmoc), 7.9 (d, 2H, H_Ar_Fmoc), 12.1 (s, 1H, CO_2_H).

*Fmoc-Glu-NH-CH_2_CH=CH_2_* (**3**): yield 90%. ^1^H-NMR (DMSO-*d*_6_): 3.7 (m, 2H, CH_2_-N), 5.0–5.2 (m, 2H, CH_2_), 5.75 (m, 1H, CH), 8.05 (t, 1H, NH). 1.8 (m, 2H, CH_2_β), 2.25 (m, 2H, CH_2_γ), 4.0 (m, 1H, CHα), 4.3 (m, 3H, 9-H, CH_2_Fmoc), 7.3 (t, 2H, H_Ar_Fmoc), 7.4 (t, 2H, H_Ar_Fmoc), 7.55 (d, 1H, NH), 7.75 (d, 2H, H_Ar_Fmoc), 7.9 (d, 2H, H_Ar_Fmoc), 12.1 (s, 1H, CO_2_H). MS 431 (M+Na^+^) found.

*Fmoc-Glu-NH-CH_2_CH_2_OH* (**4**): yield 86%. ^1^H-NMR (DMSO-*d*_6_): 3.1 (m, 2H, CH_2_-N), 3.4 (m, 2H, CH_2_-O), 4.65 (t, 1H, OH), 1.8 (m, 2H, CH_2_β), 2.2 (m, 2H, CH_2_γ), 4.0 (m, 1H, CHα), 4.2 (m, 3H, 9-H, CH_2_Fmoc), 7.32 (t, 2H, H_Ar_Fmoc), 7.41 (t, 2H, H_Ar_Fmoc), 7.55 (d, 1H, NH), 7.75 (d, 2H, H_Ar_Fmoc), 7.9 (d, 2H, H_Ar_Fmoc), 11.9 (s, 1H, CO_2_H). MS 413 (M+H^+^) found.

*Fmoc-Glu-NH-cyclohexyl* (**5**): yield 63%. ^1^H-NMR (DMSO-*d*_6_): 1.1 (m, 3H, H-hex), 1.2 (m, 3H, H-hex), 1.7 (m, 4H, H-hex), 3.5 (m, 1H, H-hex), 2.0 (m, 2H, CH_2_β), 2.2 (m, 2H, CH_2_γ), 4.0 (m, 1H, CHα), 4.2 (m, 3H, 9-H, CH_2_-Fmoc), 7.3 (t, 2H, H_Ar_-Fmoc), 7.4 (t, 2H, H_Ar_-Fmoc), 7.55 (d, 1H, NH), 7.8 (m, 3H, H_Ar_-Fmoc, NH), 7.9 (d, 2H, H_Ar_-Fmoc), 12.2 (s, 1H, CO_2_H).

*Fmoc-Glu-NH-CH_2_-cyclohexyl* (**6**): yield 53%. ^1^H-NMR (DMSO-*d*_6_): 0.8 (m, 2H, H-hex), 1.1 (m, 3H, H-Hex), 1.35 (m, 1H, H-hex), 1.65 (m, 5H, H-Hex), 2.9 (m, 2H, CH_2_-N), 7.85 (t, 1H, NH). 1.85 (m, 2H, CH_2_β), 2.25(m, 2H, CH_2_γ), 4.0 (m, 1H, CHα), 4.2 (m, 3H, 9-H, CH_2_Fmoc),7.3 (t, 2H, H_Ar_Fmoc), 7.4 (t, 2H, H_Ar_Fmoc), 7.55 (d, 1H, NH), 7.75 (d, 2H, H_Ar_Fmoc), 7.9 (d, 2H, H_Ar_Fmoc), 12.1 (s, 1H, CO_2_H).

*Fmoc-Glu-NH-CH_2_Ph* (**7**): yield 57%. ^1^H-NMR (DMSO-*d*_6_): 4.3 (m, 2H, CH_2_-N), 7.2 (m, 5H, H_Ar_), 8.45 (t, 1H, NH), 1.9 (m, 2H, CH_2_β), 2.25(m, 2H, CH_2_γ), 4.0 (m, 1H, CHα), 4.2 (m, 3H, 9-H, CH_2_Fmoc), 7.3 (t, 2H, H_Ar_Fmoc), 7.4 (t, 2H, H_Ar_Fmoc), 7.6 (d, 1H, NH), 7.75 (d, 2H, H_Ar_Fmoc), 7.9 (d, 2H, H_Ar_Fmoc), 12 (s, 1H, CO_2_H).

*Fmoc-Glu-NH-(CH_2_)_2_Ph* (**8**): yield 77%. ^1^H-NMR (DMSO-*d*_6_): 2.7 (t, 2H, CH_2_-Ar), 4.2 (m, 2H, CH_2_-N), 7.2 (m, 5H, H_Ar_), 8.0 (t, 1H, NH), 1.9 (m, 2H, CH_2_β), 2.25(m, 2H, CH_2_γ), 4.0 (m, 1H, CHα), 4.3 (m, 3H, 9-H, CH_2_-Fmoc), 7.3 (t, 2H, H_Ar_-Fmoc), 7.4 (t, 2H, H_Ar_-Fmoc), 7.5 (d, 1H, NH), 7.8 (d, 2H, H_Ar_-Fmoc), 7.9 (d, 2H, H_Ar_-Fmoc), 12.2 (s, 1H, CO_2_H).

*Fmoc-Glu-NH-(CH_2_)_3_Ph* (**9**): yield 50%. ^1^H-NMR (DMSO-*d*_6_): 1.65 (m, 2H, CH_2_), 2.6 (t, 2H, CH_2_-Ar), 3.05 (m, 2H, CH_2_-N), 7.2 (m, 5H, H_Ar_), 7.95 (t, 1H, NH), 1.95 (m, 2H, CH_2_β), 2.25(m, 2H, CH_2_γ), 4.0 (m, 1H, CHα), 4.2 (m, 3H, 9-H, CH_2_Fmoc), 7.3 (t, 2H, H_Ar_Fmoc), 7.4 (t, 2H, H_Ar_Fmoc), 7.55 (d, 1H, NH), 7.75 (d, 2H, H_Ar_Fmoc), 7.9 (d, 2H, H_Ar_Fmoc), 12.1 (s, 1H, CO_2_H).

*Fmoc-Glu-NH-CH_2_CH(Ph)_2_* (**10**): yield 71%. ^1^H-NMR (DMSO-*d*_6_): 3.6 (m, 1H, CH-Ar), 3.8 (m, 2H, CH_2_-N), 7.15 (m, 2H, H_Ar_), 7.25 (m, 8H, H_Ar_), 7.9 (t, 1H, NH). 1.65 (m, 2H, CH_2_β), 2.1 (m, 2H, CH_2_γ), 3.9 (m, 1H, CHα), 4.2 (m, 3H, 9-H, CH_2_Fmoc), 7.3 (t, 2H, H_Ar_Fmoc), 7.4 (m, 3H, H_Ar_Fmoc, NH), 7.8 (d, 2H, H_Ar_Fmoc), 7.9 (d, 2H, H_Ar_Fmoc), 12 (s, 1H, CO_2_H).

*Fmoc-Glu-NH-CH_2_-(1-naphtyl)* (**11**): yield 94%. ^1^H-NMR (DMSO-*d*_6_): 4.8 (m, 2H, CH_2_), 7.4-8 (m, 8H_Ar_-naph), 8.5 (t, 1H, NH), 1.85 (m, 2H, CH_2_β), 2.25(m, 2H, CH_2_γ), 4.0 (m, 1H, CHα), 4.2 (m, 3H, 9-H, CH_2_Fmoc), 7.3 (t, 2H, H_Ar_Fmoc), 7.4 (t, 2H, H_Ar_Fmoc), 7.55 (d, 1H, NH), 7.75 (d, 2H, H_Ar_Fmoc), 7.9 (d, 2H, H_Ar_Fmoc), 12.1 (s, 1H, CO_2_H).

*Fmoc-Glu-NH-(CH_2_)_2_(Ph(4-OH))* (**12**): yield 76%. ^1^H-NMR (DMSO-*d*_6_): 2.6 (t, 2H, CH2-Ar), 3.2 (m, 2H, CH2-N), 6.6 (d, 2H, HAr), 6.9 (d, 2H, HAr), 7.9 (t, 1H, NH), 8.2 (s, 1H, OH). 1.8 (m, 2H, CH_2_β), 2.2 (m, 2H, CH_2_γ), 4.0 (m, 1H, CHα), 4.25 (m, 3H, 9-H, CH_2_Fmoc), 7.3 (t, 2H, H_Ar_Fmoc), 7.4 (t, 2H, H_Ar_Fmoc), 7.55 (d, 1H, NH), 7.8 (d, 2H, H_Ar_Fmoc), 7.9 (d, 2H, H_Ar_Fmoc), 12 (s, 1H, CO_2_H).

*Fmoc-Glu-NH-CH_2_(Ph(3,4-OH)_2_)* (**13**): yield 78%. ^1^H-NMR (DMSO-*d*_6_): 4.1 (t, 2H, CH_2_-Ar), 6.5 (d, 1H, H_Ar_), 6.7 (m, 2H, H_Ar_), 8.25 (t, 1H, NH), 8.8 (s, 2H, 2 × OH). 1.9 (m, 2H, CH_2_β), 2.25(m, 2H, CH_2_γ), 4.0 (m, 1H, CHα), 4.25 (m, 3H, 9-H, CH_2_-Fmoc), 7.3 (t, 2H, H_Ar_Fmoc), 7.4 (t, 2H, H_Ar_Fmoc), 7.55 (d, 1H, NH), 7.8 (d, 2H, H_Ar_Fmoc), 7.9 (d, 2H, H_Ar_Fmoc), 12 (s, 1H, CO_2_H). MS 491 (M+H^+^) found.

*Fmoc-Glu-NH-CH_2_CH_2_(Ph(3,4-OH)_2_)* (**14**): yield 77%. ^1^H-NMR (DMSO-*d*_6_): 2.5 (m, 2H, CH_2_-Ar), 3.2 (m, 2H, CH_2_-N), 6.4 (d, 1H, H_Ar_), 6.6 (m, 2H, H_Ar_), 7.9 (t, 1H, NH), 8.7 (s, 2H, 2 × OH). 1.8 (m, 2H, CH_2_β), 2.2 (m, 2H, CH_2_γ), 4.0 (m, 1H, CHα), 4.25 (m, 3H, 9-H, CH_2_Fmoc), 7.3 (t, 2H, H_Ar_Fmoc), 7.4 (t, 2H, H_Ar_Fmoc), 7.55 (d, 1H, NH), 7.8 (d, 2H, H_Ar_Fmoc), 7.9 (d, 2H, H_Ar_Fmoc), 12 (s, 1H, CO_2_H). MS 505 (M+H^+^) found.

*Fmoc-Glu-NH-piperonyl* (**15**): yield 77%. ^1^H-NMR (DMSO-*d*_6_): 6.82 (d, 1H, 7'-H), 6.8 (s, 1H, 3'-H), 6.76 (d, 1H, 6'-H), 8.4 (t, 1H, NH). 1.95 (m, 2H, CH_2_β), 2.35(m, 2H, CH_2_γ), 4.0 (m, 1H, CHα), 4.3 (m, 3H, 9-H, CH_2_Fmoc), 7.3 (t, 2H, H_Ar_Fmoc), 7.4 (t, 2H, H_Ar_Fmoc), 7.6 (d, 1H, NH), 7.75 (d, 2H, H_Ar_Fmoc), 7.9 (d, 2H, H_Ar_Fmoc), 12 (s, 1H, CO_2_H). 

*Fmoc-Glu-NH-(8-coumarinyl)* (**16**): yield 47%. ^1^H-NMR (DMSO-*d*_6_): 7.7 (m, 3H, 4',5',7'-H), 10.3 (s, 1H, NH), 7.4 (m, 1H, 8'-H), 6.5 (d, 1H, 3'-H). 1.95 (m, 2H, CH_2_β), 2.25(m, 2H, CH_2_γ), 4.0 (m, 1H, CHα), 4.3 (m, 3H, 9-H, CH_2_Fmoc), 7.3 (t, 2H, H_Ar_Fmoc), 7.4 (t, 2H, H_Ar_Fmoc), 7.6 (d, 1H, NH), 7.9 (d, 2H, H_Ar_Fmoc), 8.1 (d, 2H, H_Ar_Fmoc), 12 (s, 1H, CO_2_H).

*Fmoc-Glu-NH-CH_2_-4-(7-MeO-coumarinyl)* (**17**): yield 74%. ^1^H-NMR (DMSO-*d*_6_): 3.8 (s, 3H, CH_3_O), 4.5 (m, 2H, 4'-CH_2_), 6.15 (s, 1H, 3'-H), 6.92, 6.95 (dd, 1H, 6'-H), 7.0 (d, 1H, 8'-H), 7.7 (m, 1H, 5'-H), 8.5 (t, 1H, NH). 1.9 (m, 2H, CH_2_β), 2.3 (m, 2H, CH_2_γ), 4.1 (m, 1H, CHα), 4.3 (m, 3H, 9-H, CH_2_Fmoc), 7.3 (t, 2H, H_Ar_Fmoc), 7.4 (t, 2H, H_Ar_Fmoc), 7.6 (d, 1H, NH), 7.7 (d, 2H, H_Ar_Fmoc), 7.9 (d, 2H, H_Ar_Fmoc), 12 (s, 1H, CO_2_H).

### 3.3. New Synthesis of Reference Peptide **2**

Starting from Rink amide MBHA resin (200 mg, 0.45 mmol/g), the synthesis of linear peptides were conducted by CEM-Liberty 1 synthesizer with Fmoc chemistry at 0.1 mmol scale. The coupling was realized at 50 °C for 10 min with microwave irradiation, with HBTU/DIEA as coupling reagents. Fmoc deprotection was conducted at 50 °C for 5 min. Then the peptidyl resin was placed in a syringe adapted with a frit and a stopper. After the washing steps with CH_2_Cl_2_, a solution of 2% TFA and 5% TIPS in CH_2_Cl_2_ (20 mL in total) was added, and the syringe was shaken for 5 min before draining. This procedure was repeated one time to insure complete deprotection. Then the resin was washed with CH_2_Cl_2_ and swollen in DMF (5 mL). HBTU/HOBt/DIEA (1.5/1.5/4.5 equivalents to resin loading) were added and the resulting suspension was shaken overnight on a wheel. After draining and washing steps with DMF and CH_2_Cl_2_, the peptidyl resin was dried and cleaved by a solution of 2.5% TIPS and 2.5% water in TFA for 2 h. The resin was then removed by filtration and the filtrate was condensed by evaporation. The residue was precipitated in ether and centrifuged. The precipitate was washed two times with ether and collected by centrifugation. The crude cyclic peptide was then purified by semi-preparative HPLC. The fractions were checked by analytical HPLC analysis, collected and lyophilized. The peptide identity was confirmed by mass spectrometry analysis.

### 3.4. Synthesis of C-Terminal Substituted Cyclic Peptides (Peptides **3**–**19**)

Starting from Fmoc-Gly-Cl Trt resin, the synthesis of linear peptides were conducted on a CEM-Liberty 1 synthesizer with Fmoc chemistry at 0.1 mmol scale. The coupling was realized at 50 °C for 10 min with microwave irradiation, with HBTU/DIEA as coupling reagents. Fmoc deprotection was conducted at 50 °C for 5 min. The linear peptide was then cleaved from resin by treatment with 2% TFA and 5% TIPS in CH_2_Cl_2_ (10 mL) during 1 h. The suspension was filtered to 10% pyridine methanol solution (4 mL). After solvents evaporation, the residue was triturated with water and the precipitate collected and dried to give the side chain protected linear peptide. This crude peptide was then dissolved in 50 mL of DMF, HOAt (41 mg, 0.3 mmol) and DIC (46 µL, 0.3 mmol) were added and the mixture was stirred at room temperature during 1–3 days following HPLC check. The color change of HOAt, yellow-colorless-yellow, helped to indicate also the completion of the cyclisation. DMF was then removed by evaporation and the residue precipitated in water, washed thoroughly with an aqueous NaHCO_3_ solution to remove diisopropylurea and HOAt, and dried. The crude cyclic protected peptide was then treated with 50% TFA in CH_2_Cl_2_ (10 mL in total) with 2% TIPS during 2 h. After evaporation, the residue was precipitated in ether and centrifuged. The precipitate was washed two times with ether and collected by centrifugation. The crude cyclic peptide was then purified by semi-preparative HPLC. The fractions were checked by analytical HPLC analysis, collected, and lyophilized. The peptide identity was finally confirmed by mass spectrometry analysis. Analytical results are represented in Table 3.

**Table 3:** Cyclic peptides with C-terminal modifications. Yield is the total yield of linear peptide synthesis and its cyclization. MS is obtained by ESI^+^ method. HPLC retention times (Rt) obtained by the gradient indicated (mobile phases A: 0.1% TFA aqueous; B: 0.09% TFA in 70% acetonitrile aqueous solutions). All numbers of peptides refers to Table 1.

Peptide	Yield (%)	MS Found	Rt (Minutes)
**2**	10 (8.8 [26])	963 (M+H^+^)	12.5 (10%–60% B in 30 min)
**3**	29.0	1034 (M+H^+^)	14.8 (20%–80% B in 30 min)
**4**	12.7	1020 (M+H^+^)	15.1 (20%–80% B in 30 min)
**5**	3.5	1004 (M+H^+^)	10.2 (20%–70% B in 20 min)
**6**	7.8	1008 (M+H^+^)	11.0 (10%–60% B in 20 min)
**7**	23.4	1046 (M+H^+^)	18.0 (10%–60% B in 20 min)
**8**	10.5	1060 (M+H^+^)	15.4 (20%–70% B in 20 min)
**9**	6.5	1054 (M+H^+^)	13.0 (20%–70% B in 20 min)
**10**	30.9	1067 (M+H^+^)	14.0 (20%–80% B in 30 min)
**11**	15.5	1081 (M+H^+^)	15.7 (20%–70% B in 20 min)
**12**	11.8	1144 (M+H^+^)	17.9 (20%–70% B in 20 min)
**13**	37	1125 (M+Na^+^)	18.5 (20%–80% B in 30 min)
**14**	20.3	1083 (M+H^+^)	13.7 (20%–80% B in 30 min)
**15**	11.1	1085 (M+H^+^)	13.5 (10%–60% B in 20 min)
**16**	30.8	1099 (M+H^+^)	14.4 (10%–60% B in 20 min)
**17**	29.2	1098 (M+H^+^)	12.8 (20%–70% B in 20 min)
**18**	3.3	1108 (M+H^+^)	12.2 (20%–70% B in 20 min)
**19**	26.6	1152 (M+H^+^)	13.5 (20%–70% B in 20 min)

### 3.5. ELISA VEGF-VEGFR1 Binding Inhibition Assay

The 96-well plates were coated with humanized extracellular domains (ECD) of VEGFR-1 (R&D Systems, Abingdon, UK) in PBS (20 ng/well) overnight at 4 °C. On the following day, the plates were washed with 250 µL wash buffer (PBS containing 0.1% (v/v) Tween 20) three times and treated with blocking buffer (PBS containing 3% (w/v) BSA) at 37 °C for 2 h, followed by three washes with wash buffer. 50 µL of peptides solution at twice the desired final concentration (in PBS containing 2% DMSO) were added in triplicate wells and the plate was kept at 37 °C for 1 h. A solution of btVEGF-A_165_ (R & D Systems, Abingdon, UK) at twice the desired final concentration (typically 100 pM) in 50 µL PBS was added. After 2 h incubation, the plates were washed four times with wash buffer. 100 µL of Streptavidin‑Horseradish Peroxidase (Amersham, Pittsburgh, PA, USA) diluted 1:8000 w in PBS containing 0.1% (v/v) Tween 20 and 0.3% (w/v) BSA) were then added to each well to detect the btVEGF‑A_165_ bound to the ECD of VEGFR1. After 45 min incubation at 37 °C and in the dark, the plate was washed five times with wash buffer. A volume of 100 µL of SuperSignal West Pico Chemioluminescent Substrate (Pierce, Rockford, IL, USA) was added and the chemiluminescence was quantified with a Perkin Elmer Victor 2 spectrophotometer (Victor Wallac Multilabel reader). The percentages of displacement were calculated by the following formula: 100 × [1 − (S − NS)/(MS − NS)] where S is the signal measured, NS is the nonspecific binding signal defined as the signal measured in the absence of coated receptor on the microplate, and MS is the maximum binding signal obtained with (bt)-VEGF-A_165_ without competitor.

## 4. Conclusions

We described here the optimization of a cyclic peptide developed in our laboratory. The best peptides are in further cellular assays to evaluate their anti-angiogenic abilities. In particular, peptide **19** carrying a fluorescent coumarin group can be used as a biological marker tool for imaging applications, and could also be useful for X-ray co-structure studies.
